# AGING DIMINISHES THE DIRECT ASSOCIATION BETWEEN BRAIN ACTIVATION AND POSTURAL CONTROL DURING THE N-BACK TASK

**DOI:** 10.1093/geroni/igz038.3437

**Published:** 2019-11-08

**Authors:** Azizah J Jor'dan, Ikechukwu Iloputaife, Brad Manor

**Affiliations:** 1 VA Boston Healthcare System, Boston, Massachusetts, United States; 2 Hebrew SeniorLife, Marcus Institute for Aging Research, Boston, Massachusetts, United States; 3 Hinda and Arthur Marcus Institute for Aging Research, Harvard Medical School, Boston, Massachusetts, United States

## Abstract

Aging diminishes the control of standing posture, which relies upon the capacity to activate brain regions involved in cognitive-motor function. Our prior work shows that impaired cerebral blood flow (CBF) regulation within the middle cerebral artery (MCA) in response to the N-Back executive function task is linked to worse walking performance in older adults. However, the effects of aging on the relationship between CBF regulation and postural control during the N-Back task is unknown. Sixteen young (27 years) and 15 older participants (76 years) stood upright and completed the N-Back (i.e., control [Identify X, IdX] and an experimental condition [2-Back]) presented on a screen while CBF and postural sway were simultaneously recorded. CBF was recorded using transcranial Doppler. Sway was recorded using a lumbar motion sensor. Elliptical area, root mean square (RMS), distance, velocity, and acceleration were computed. There were no group differences in sway outcomes (p>0.37). Young participants had higher CBF during the IdX and 2-Back compared to older participants (p<0.001). Within the young, and not within older participants, those with lower CBF during performance of the 2-Back exhibited greater elliptical area (β=-0.67, p=0.03) and RMS (β=-0.68, p=0.03), faster acceleration (β=-0.78, p=0.02), and longer distance (β=-0.64, p=0.04). There were no associations between CBF and sway outcomes in either group during the IdX. These results suggest that dual-task sway performance is directly linked to CBF in younger adults, and furthermore, this link is diminished in older adults. These results underpin the need for cognitive-motor interventions in older adults.

